# Adaptation of the Pediatric Smell Wheel^TM^ to evaluate olfactory function in Brazilian children

**DOI:** 10.1016/j.bjorl.2021.08.004

**Published:** 2021-10-17

**Authors:** Marco A. Fornazieri, Lucas K. Ebara, Rafael Goulart de Araújo, João Vitor Fernandes Lima, Felipe B. Favareto, Fábio R. Pinna, Richard L. Voegels, Richard L. Doty

**Affiliations:** a Universidade Estadual de Londrina (UEL), Departamento de Cirurgia Cliníca, Londrina, PR, Brazil; b Pontifícia Universidade Católica do Paraná (PUC PR), Departamento de Medicina, Londrina, PR, Brazil; c Universidade de São Paulo (USP), Departamento de Otorrinolaringolgia, São Paulo, SP, Brazil; d University of Pennsylvania, Perelman School of Medicine, Smell and Test Center, Department of Otorhinolaryngology – Head and Neck Surgery, Pennsylvania, USA

**Keywords:** Adenoids, Diagnostic tests, Smell, Olfaction disorders, Nasopharyngeal diseases

## Abstract

**Objective:**

Olfaction influences nutrition, safety, and the cognitive development of children. Presently there are few olfactory tests for children, and normative reference values are generally lacking. In this study, we validate the Pediatric Smell Wheel^TM^ (PSW) to evaluate olfactory function in Brazilian children.

**Methods:**

We modified and validated the PSW, a test developed in the United States, for administration in Brazil, and established normative data for Brazilian children. The validation process consisted of five phases. First, we identified odorants familiar to Brazilian children. Second, we established the test-retest reliability of the Brazilian PSW test that employed these odorants. Third, we compared the test scores of healthy children to those microsmic from adenoid hypertrophy. Fourth, we examined the test’s ability to detect improvement in olfactory function before and after adenoidectomy. Finally, we determined a normative 10th percentile cut point for defining microsmia in a sample of 169 healthy Brazilian children between 5 and 12 years of age.

**Results:**

Eleven odors were identified for inclusion in the test. The test-retest reliability was modest (r = 0.54, *p* < 0.001), although likely attenuated based on the inclusion of only data from children with normal function. The test clearly differentiated the olfactory function of children with adenoid hypertrophy from that of healthy children and demonstrated the return of function after adenoidectomy. An overall score <7 was found useful in defining hyposmia, although additional age-related cut-points were also defined.

**Conclusion:**

A Brazilian version of the PSW was developed and validated and found to be efficacious in detecting smell dysfunction in children ranging in age from 5 to 12 years.

**Level of evidence:**

1b (Diagnosis).

## Highlights

• This study developed and validated the Pediatric Smell Wheel™ (PSW) for testing Brazilian children.
• The Brazilian PSW differentiated the olfactory function of children with adenoid hypertrophy from that of healthy children.
• A score below 7 out of 11 is a useful cutoff for defining microsmia in young children.

## Introduction

One of the difficulties when performing an olfactory examination on a child is to hold their attention. In 2012, Cameron and Doty published the first paper on the development of the Pediatric Smell Wheel^TM^ (PSW), an olfactory identification test with 11 odors applicable to the pediatric population.^1^ The stimuli of this test are contained in plastic microcapsules positioned along the circumference of a cardboard disk. The disk rotates within an outer jacket, such that only one “scratch and sniff” odorant patch and four response alternatives are exposed at a time in a small window of the outer jacket for sampling. The examiner releases the odor by scraping the strip with a pencil tip and presenting it to the child at a distance of ∼1 cm from the nares. Older children can perform this themselves without help of an adult. Once an odorant is smelled, the child indicates which of four named and pictured alternatives best describes the smell. A response is required even if no smell is perceived, i.e., the test is a forced-choice test. The disk is then rotated to the next odorant. Based on the total number of correct responses, the child’s function can be classified as either normal or abnormal for an individual of his or her age.

The PSW has the advantage of being like a children's game in which the child, when identifying one of the smells by pictures or words, turns the wheel to the next step of the test as if it were a new challenge. Although there are other validated olfactory tests for children,^2^ only the PSW has a playful aspect that enhances the child’s cooperation in carrying out the test. Knowing a child’s olfactory capacity is essential because the sense of smell influences their perception and appreciation of food,^3^, ^4^ sensorial development,^5^ safety, and hygiene.^6^ Unfortunately, many parents are unaware of their child’s olfactory disorder until adolescence, which can delay the diagnosis of, and treatment for, the endocrine disturbances that accompany congenital anosmia’s such as Kallmann’s syndrome.^7^

International versions of odor identification tests such as the PSW require that the involved odorants are applicable to the countries in which they are administered. This reflects the fact that familiarity with odors can vary across cultures, particularly among children who have not experienced the same range of odors as adults.^8^, ^9^ The validation process for adapting a test to a given culture consists of several phases,^2^ including the choice of familiar odors, determining the ability of the test to differentiate healthy children from those with olfactory dysfunction, and establishing normative values for the test.

In this study, we describe the adaptation and validation of the PSW (Sensonics International, Haddon Heights, NJ 08035) to the Brazilian pediatric population employing these principles.

## Methods

### Subjects

Two hundred and sixteen Brazilian children, 130 boys and 86 girls, between 5 and 12 years old were recruited. One hundred sixty-nine were students from a public school whose parents did not report any difficulties of their children to smell or taste food, and who had no previous histories of head trauma, mouth breathing, nasal diseases, or current upper respiratory tract infections. The other 47 participants were children in a waiting list for adenoidectomy in an outpatient otolaryngology clinic, without a previous history of head trauma. Signed informed consent was obtained from the parents or responsible guardian. The study was approved by the local ethics committee (CAAE: 21097413.3.0000.5231).

### Validation process

In an earlier pilot study, eleven odors that had been easily identified by Brazilian adults in an validation study of the University of Pennsylvania Smell Identification Test for Brazil^9^ were administered to sixty children of the same age group as those of the present study. In this preliminary study, the odorants were onion, soap, tire, chewing gum, banana, cherry, baby powder, chocolate, smoke, mint, and cinnamon. Since three odors, namely banana, cherry, and chocolate, were misidentified by more than half of the subjects, they were replaced with the odors of grape, flowers, and peanut, respectively; the final odors employed in the Brazilian PSW administered to the 216 children of this study are shown in Table 1.

**Table 1:** Odorants and alternatives in the adapted Smell Wheel for the Brazilian pediatric population.

Odorants English/Portuguese	Alternative A	Alternative B	Alternative C	Alternative D
Onion	Onion	Chocolate	Watermelon	Banana
Cebola	Cebola	Chocolate	Melancia	Banana
Soap	Soap	Fish	Chocolate	Peanut
Sabonete	Sabonete	Peixe	Chocolate	Amendoim
Tire	Tire	Watermelon	Banana	Gingerbread
Pneu	Pneu	Melancia	Banana	Pão de mel
Bubblegum	Smoke	Skunk	Bubblegum	Onion
Chiclete	Fumaça	Gambá	Chiclete	Cebola
Grape	Grape	Pizza	Pasta	Mint
Uva	Uva	Pizza	Macarrão	Hortelã
Flower	Flower	Gingerbread	Apple	Strawberry
Flor	Flor	Pão de mel	Maçã	Morango
Baby Powder	Babe powder	Pineapple	Cheese	Cherry
Talco de bebê	Talco	Abacaxi	Queijo	Cereja
Peanut	Garlic	Peanut	Orange	Tire
Amendoim	Alho	Amendoim	Laranja	Pneu
Smoke	Apple	Grass	Smoke	Grape
Fumaça	Maçã	Grama	Fumaça	Uva
Wintergreen	Tomato	Wintergreen	Strawberry	Honey
Menta	Tomate	Menta	Morango	Mel
Cinnamon	Watermelon	Cinnamon	Smoke	Coconut
Canela	Melancia	Canela	Fumaça	Coco

Of these children, 34 were tested again on a second occasion 15–60 days later to establish the test’s test-retest reliability. Moreover, the sensitivity of the test in differentiating healthy children from those with known olfactory dysfunction secondary to marked adenoid hypertrophy in which more than 50% of the rhinopharynx^10^ was occupied was assessed. Subsequently, it was determined whether the test scores of these children returned to normal a month or more following surgical removal of their adenoids. Based on the data collected in this study, a normative cut-off points at the level of the 10th percentile for establishing microsmia was set, i.e., a score equal or lower than this value would classify the child as having olfactory dysfunction.^11^

### Statistical analyses

The percentages of correct answers in identifying children's odors were determined together with their respective 95% confidence intervals. The normality of the data was analyzed by the Shapiro-Wilk test, followed by the Student’s *t*-test to compare the olfactory function scores between the different groups of children. Pearson’s correlation was applied to analyze test-retest reliability. The scoring difference between boys and girls was analyzed by multiple linear regression in healthy children, with the Smell Wheel score as the dependent variable and gender and age as independent variables. Statistical analysis was performed using the software Stata 13.0 (StataCorp., Texas, USA).

## Results

The demographic characteristics of the tested children are described in Table 2. The number of correct responses given to each odorant by the healthy children is shown in Table 3. The data from the healthy children who were unable to identify at least 5 of the 11 smells of the olfactory test were excluded from the overall analyses, including the development of the normative data set, since the probability of an underlying disease affecting olfaction not cited or not known by the parents existed (n = 16).

**Table 2:** Demographic characteristics of the study children.

Variables	Normal children (n = 153)	Children with adenoid hypertrophy (n = 47)
Age (mean, SD)	8.4 (2.2)	6.9 (2.0)
Sex (% male)	54.6	65.9
Race (%)		
White	71.4	68.9
Non-White	28.6	31.1

**Table 3:** Percentage of correct answers in the Brazilian Smell Wheel in children without nasal or olfactory complaints.

Odor	Normal children (%, 95% CI) (n = 153)
Onion	83.0 (77.0–89.0)
Soap	92.8 (88.7–97.0)
Tire	71.2 (64.0–78.5)
Bubblegum	81.1 (74.8–87.3)
Grape	47.1 (39.1–55.1)
Flower	75.2 (68.2–82.1)
Baby powder	79.1 (72.6–85.6)
Peanut	41.8 (33.9–49.7)
Smoke	77.6 (70.9–84.3)
Wintergreen	75.2 (68.2–82.1)
Cinnamon	76.5 (69.7–83.3)

The most identified odor was soap (92% of correct answers) and the most erroneously identified odor was peanut (41.8%). The test scores of the 34 healthy children who took the test twice did not differ significantly on the two test occasions (respective means [SDs] = 8.6 [1.7] & 8.9 [1.5], *p* = 0.23) Although the test-retest reliability was moderate (r = 0.54, *p* < 0.001), this value was attenuated by the lack of inclusion of data from smell-compromised subjects, thereby limiting the range of test scores upon which a correlation coefficient depends.

**Figure 1:**
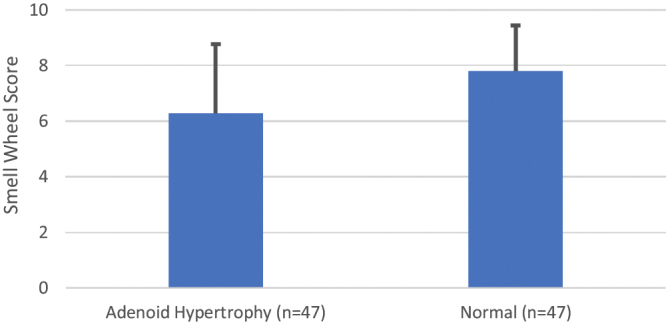
Pediatric Smell Wheel score means and standard deviations among children with and without adenoid hypertrophy matched for age, sex, and ethnicity (*p* < 0.001).

The test clearly differentiated the olfactory function of children with severe adenoid hypertrophy from that of healthy children matched on the basis of sex, race, and age (Fig. 1), and demonstrated the return of function after the surgery. Thus, the post-operative PSW score improved significantly after the adenoidectomy (n = 47, preoperative score = 6.3 [SD = 2.5] and postoperative score = 9.3 [SD = 1.2], *p* < 0.001). The value of 6 was found to fall at the 10th percentile of the normative data that were collected. Thus, children from 5 to 12 years of age who score less than 7 out of 11 questions on the Brazilian PSW can be considered microsmic. There was a statistically significant correlation between test scores and age (r = 0.31, *p* < 0.001). Therefore, based upon this association and the 10th percentile, additional age-related cut-points were defined; namely, 5 for children less than eight years of age, 6 for those between 8 and 10 years of age, and 7 for those more than ten years old. There was no difference in scores between boys and girls (*p* = 0.29), making it unnecessary to obtain different normality thresholds according to sex.

## Discussion

By employing a large sample of children 5–12 years of age and a defined set of validation procedures, this study developed and validated a version of the Pediatric Smell Wheel™ (PSW) for administration in the Brazilian population. The test was sensitive and capable of clearly differentiating the olfactory function of children with adenoid hypertrophy from that of healthy children. Moreover, it was able to demonstrate the return of function after adenoidectomy. Useful cut-points for defining smell loss were established.

In this study, even after choosing and changing odors according to the most correctly identified ones in previous studies, two odors remained with an accuracy rate below 50%. Compared to other olfactory test development studies, this rate is low. However, the requirement of high identification rates for all odorants in a test is not sancrosanct, and it is unknown whether having a two odors with somewhat lower identification significantly influences testing capabilities of an 11-odor test.^12^ The current version of the adapted PSW™ easily differentiated healthy children from those with an olfactory deficit, validating its clinical utility. Whether further modifications, aside from significantly increasing the total number of test items, would meaningfully improve such utility is unknown.

The present study suggests that, from both a heuristic and empirical perspective, a score below 7 is useful for defining microsmia in young children. Although one has to be aware that cut-off points have some degree of arbitrariness and there is considerable variability among individuals around such points, cut points can be further defined relative to the needs of the examiner and the ages of the involved subjects. Based upon the 10th percentile, the present study established a cut-point at 5 for children less than eight years of age, 6 for those between 8 and 10 years of age, and 7 for those more than ten years old. These cut-points may be of particularly of value in research where group averages are involved, and increased measurement sensitivity is desired.

Boys and girls achieved similar scores in our sample. In adults, it is well known that women outperform men on more extensive olfactory tests (e.g., the 40-item University of Pennsylvania Smell Identification Test or UPSIT®), although there is considerable overlap and the largest sex differences appear after the age of 65 years.^13^, ^14^ Nonetheless, girls before the age of puberty outperform boys of the same age on such tests,^15^, ^16^ suggesting that brief tests likely lack the statistical power to detect this sex difference. Such findings, along with studies of post-menopausal women,^16^ suggest that concurrent gonadal hormones are not the cause of the sex difference.^15^

This study has both strengths and weaknesses. Among its strengths are its relatively large sample size, its inclusion of both sexes, and its sensitivity to nasal conditions known to impact smell function. Among its limitations are the inclusion of subjects from only one country, the omission of subjects with a wide range of olfactory deficits, and the inclusion of two odors with a lower than desired identification rate. However, as noted above, such a rate is somewhat arbitrary, and their inclusion is unlikely to significantly limit the clinical utility of the test, particularly in light of its demonstrated ability to clearly differentiate children with normal and abnormal smell function.

## Conclusions

The PSW is now adapted for application in Brazilian children, and the method used can be used in other countries for its adaptation. This test is another option for a more accurate examination of children’s olfactory function.

## Conflicts of interest

RLD is a consultant to Eisai Co, Ltd, Merck, the Michael J. Fox Foundation for Parkinson’s Disease Research, and Johnson & Johnson. He receives royalties from Cambridge University Press, Johns Hopkins University Press, and John Wiley & Sons, Inc. He is president of, and a major shareholder in, Sensonics International, a manufacturer and distributor of smell and taste tests, including the test used in this study.
